# Towards a subsiding diabetes epidemic: trends from a large population-based study in Israel

**DOI:** 10.1186/s12963-014-0032-y

**Published:** 2014-10-30

**Authors:** Tomas Karpati, Chandra J Cohen-Stavi, Morton Leibowitz, Moshe Hoshen, Becca S Feldman, Ran D Balicer

**Affiliations:** Clalit Research Institute, Chief Physician’s Office, Clalit Health Services, 101 Arlozorov Street, 62098 Tel Aviv, Israel; Department of Medicine, New York University School of Medicine, New York, USA; Department of Public Health, |Ben-Gurion University of the Negev, Beer-Sheva, Israel

**Keywords:** Diabetes epidemiology, Diabetes prevalence, Diabetes incidence, Diabetes mortality, Population at risk, Diabetes screening rates

## Abstract

**Background:**

With increasing diabetes prevalence worldwide, an impending diabetes “pandemic” has been reported. However, definitions of incident cases and the population at risk remain varied and ambiguous. This study analyzed trends in mortality and screening that contribute to diabetes prevalence and incidence, distinguishing between new incident cases and newly detected cases.

**Methods:**

In an integrated provider-and-payer-system covering 53% of Israel’s population, a composite diabetes case-finding algorithm was built using diagnoses, lab tests, and antidiabetic medication purchases from the organization’s electronic medical record database. Data were extracted on adult members aged 26+ each year from January 1, 2004 through December 31, 2012. Rates of diabetes prevalence, incidence, screening, and mortality were reported, with incidence rates evaluated among the total, “previously-screened,” and “previously-unscreened” at-risk populations.

**Results:**

There were 343,554 diabetes cases in 2012 (14.4%) out of 2,379,712 members aged 26+. A consistent but decelerating upward trend in diabetes prevalence was observed from 2004–2012. Annual mortality rates among diabetics decreased from 13.8/1000 to 10.7/1000 (p = 0.0002). Total population incidence rates declined from 13.3/1000 in 2006 to 10.8/1000 in 2012 (p < 0.0001), with similar incidence trends (13.2/1000 to 10.2/1000; p = 0.0007) among previously-screened at-risk members, and a rise in testing rates from 53.0% to 66.7% (p = 0.0004). The previously-unscreened group decreased 28.6%, and the incidence rates within this group remained stable.

**Conclusions:**

The increase in diabetes prevalence is decelerating despite declining mortality and increasing testing rates. A decline in previously-screened incident cases and a shrinking pool of previously-unscreened members suggests that diabetes trends in Israel are moving toward equilibrium, rather than a growing epidemic.

**Electronic supplementary material:**

The online version of this article (doi:10.1186/s12963-014-0032-y) contains supplementary material, which is available to authorized users.

## Background

Diabetes mellitus is a chronic illness that contributes greatly to overall morbidity and mortality and has been widely reported to be increasing in prevalence throughout the world [1]. There have been myriad efforts to uncover the causes contributing to this trend, including decreasing mortality rates and resultant aging of the population, increasing incidence rates, and more effective detection of diabetes [2-8]. The Centers for Disease Control and Prevention (CDC) utilizing diagnoses of diabetes obtained from the 2005–2008 National Health and Nutrition Examination Survey (NHANES) reports significant increases in both prevalence and incidence of diabetes in the United States from 1992 to 2010 [9]. Boyle et al. using US population statistics predicts that by 2050, 21% of the population will be diabetic [10]. The global burden and emerging pandemic of diabetes has been related to increases in obesity, decreases in physical activity, and the aging of the population [9,11].

Accurate assessment of prevalence and incidence of diabetes, however, is very much dependent on the data used to evaluate these trends [12]. In 2012, the London School of Economics carried out a survey in five European countries (Italy, Germany, Spain, United Kingdom, and France) and reported a paucity of reliable data for national registries of diabetes incidence and prevalence [13]. Nevertheless, that survey reported that there was growing concern in these countries related to increasing incidence. Well documented diabetes registries from limited localities in Taiwan, Canada, Denmark, Scotland, UK, and Italy document a consistent annual increase in prevalence in all cases, with most also reporting rising incidence [2,5-7,14,15].

A recent review of conceptual and practical challenges to the development of diabetes registries highlighted issues of case definition particularly in instances of uncertain gold standards [12]. The authors make the point that multiple inclusion criteria have to be incorporated into the case definition to assure high sensitivity in case identification but should also allow for flexibility by applying more stringent criteria to account for considerations of specificity. In almost all efforts to capture diabetes incidence and prevalence, there has been an integration of any number of different datasets dependent on the constructs of local data availability and definitions. These include varying combinations of hospital discharge summaries, outpatient medical records, laboratory test results, and medications dispensed. Several large studies have used data on diagnoses and medications from medical records, claims-based data, and pharmacy records [2,7,14]. A few relatively smaller studies have also had access to data capturing the date of diagnostic or screening tests (glucose or HbA1c) and clinical laboratory results [4,5,15]. These limitations in access to data impact how diabetes is defined and how new incident cases are identified.

Defining the prevalence and incidence of diabetes is particularly challenging, because it is a chronic illness with an indeterminate latent phase that is often asymptomatic and may go undiagnosed. Therefore, screening and detection efforts play a part in how the onset of disease and incidence are defined; however, most studies reporting on diabetes prevalence and incidence do not examine screening policies or detection rates as part of their assessments. In measuring annual incidence rates, a key point is how the *population at risk* is characterized. One characterization is to take the entire population without a previous diagnosis of diabetes and by the enumeration of new diagnoses, measure new cases. This approach does not make a distinction between patients who have just developed the disease and patients who have had the disease for some time but were only recently tested for the first time and thus, the disease has just been detected and labeled as “newly-detected cases”. Previous research examining diabetes prevalence and incidence does not distinguish between “new incident” cases and “newly-detected” cases in defining their population at risk, thereby resulting in ambiguous representations of incidence.

The present study was undertaken to disentangle the complexity of factors contributing to diabetes prevalence, incidence, and mortality trends in a large, closed payer-provider health system between 2004 and 2012. In documenting diabetes prevalence and incidence trends, our study is the first to examine screening rates and use a screening-based incidence measure to distinguish between “new incident” cases and “newly-detected” cases. In parallel, to insure full and accurate enumeration of the diabetes cases in our adult population, an algorithm incorporating six sets of criteria derived from a large, integrated clinical and administrative data warehouse was created and utilized for case identification.

## Methods

### Data sources and study population

Clalit Health Services (Clalit), the largest integrated health care service provider and payer system in Israel, has a comprehensive health care data warehouse which combines hospital and community medical records, laboratory and imaging information, pharmaceutical records, and Ministry of the Interior vital statistics. There is universal health care coverage in Israel, and membership turnover within Clalit is less than 1% annually [16], facilitating the study of population trends over time. The Clalit membership includes a relatively larger proportion of minority (27% vs. 21%) and lower socio-economic individuals compared to the general Israel population, with 39.8% of Clalit’s adult population earning minimum wage or below in 2011 compared to 38.8% in the overall population [17]. For the current study, diagnosis information from patient electronic medical records (EMR), blood test results from laboratory records, dispensed medications from pharmacy records, age and sex data from demographic records, and mortality statistics were accessed on Clalit’s diverse population of over four million patients, which comprise more than half of the Israeli population. An open cohort was created based on data extracted on all the adult members aged 26 and older during each year from January 1, 2004 through December 31, 2012. The study and use of this data was approved by Clalit’s institutional review board.

### Diabetes case finding

An algorithm was developed incorporating four parameters (HbA1c tests, glucoses tests, diagnoses, and diabetes medications) into six diagnostic criteria to create a composite definition of diabetes mellitus for case identification (Additional file 1: Figure S1). An internal validation of this algorithm and further elaboration on the derivation of the composite algorithm are presented in Additional file 2: Table S1. Excluded from the algorithm were those who only had gestational diabetes and did not meet the diabetes criteria prior to or subsequent to pregnancy. As there is no documentation for the week of pregnancy, the pregnancy period was determined as the 42 weeks before delivery (when a delivery data was available), 20 weeks before an abortion, or nine months before and six months after a diagnosis of pregnancy, when indicated. If a diabetes criterion was identified for the first time during pregnancy, the patient was indicated as having gestational diabetes and subsequently excluded from the study’s population. Additionally, no distinction could be made between type 1 and type 2 diabetes; however, by limiting the population studied to adults aged 26 and older, we minimize the inclusion of incident type 1 cases.

Annual diabetes diagnoses from both hospital and community records (ICD-9 codes 250 and all its subordinate codes 250.x), lab tests from community facilities, and antidiabetic medication purchases were compiled to build the composite algorithm comprised of the following six internally validated diagnostic criteria: 1) *Lab Tests* - Glucose ≥11.1 mmol/l (200 mg/dl) once; 2) HbA1c >7.0 once; 3) HbA1c ≥6.5 with a glucose test ≥7.0 mmol/l (126 mg/dl), 12 months before or three months after the HbA1c test date; 4) *Diagnosis + Lab test* - Diabetes diagnoses (ICD-9 codes 250 and 250.x) with a lab test confirmation (Glucose ≥7.0 mmol/l (126 mg/dl) or HbA1c ≥6.5) within a period of 12 months before the diagnosis or up to three months after the diagnosis date; 5) *Medication + Lab test-* Hypoglycemic drug (Anatomical Therapeutic Chemical classification level 3 codes A10B and A10A) purchase and a lab test confirmation within a period of 12 months before the purchase or up to three month after the purchase date (≥7.0 mmol/l (126 mg/dl) or a HbA1c ≥6.5); 6) *Diagnosis + Medication* - diabetes diagnosis and a concomitant antidiabetic drug purchase within a period of 12 months before and three months after the diagnosis date. The date of diabetes registry entrance was determined by the earliest date for which one or more of the algorithm composite criteria were met (Additional file 3: Table S2).

### Analysis

Trends in diabetes prevalence, incidence, screening, and mortality were reported as crude and age-standardized rates, using the 2005 overall age distribution of the 26 and older Clalit population as the standard. Annual prevalence, incidence, and mortality rates were also stratified by 10-year age groups.

*Prevalence* was defined as the annual number of diabetes patients at the end of each calendar year, using the Clalit composite definition of diabetes, divided by the total Clalit population (26+) alive at the end of each calendar year, reported per 1,000 members.

*Mortality* rates of members with diabetes were reported annually as a proportion of the population with diabetes who died over the course of the year out of those who had diabetes at the beginning of the year.

*Diabetes screening rates* were evaluated annually, defined as members who had a recorded glucose or HbA1c test anytime during a given year, divided by the total non-diabetic population 26 years and older at the beginning of that year, who were not screened in the previous three years. We used a three-year screening period because this is the recommended time indicated in the US guidelines for diabetes screening [18]. The unscreened population was also examined, defined as those members without diabetes who did not have a glucose or HbA1c test in the previous three years.

For capturing incidence, we examined the total population at risk (without diabetes at the beginning of the year), as well as distinguished between members at risk who had or had not been screened within the previous three years. Total population incidence was reported as the annual new cases of diabetes by the end of the year, divided by the total population at-risk at the beginning of each calendar year. Those members that were first identified as having diabetes and who died during the same year were not included as cases in the numerator. *“Previously-screened incidence”* was defined as new cases in each calendar year between 2006–2012 among only the members who received a glucose or HbA1c test (“screened”) at least once in the previous three years and who had no indication of diabetes at the beginning of the year. This was to distinguish patients who had not undergone tests to detect diabetes in recent years, and thus, whose diabetes status was not clearly determined at the beginning of that year, from patients who were recently screened by the beginning of the year and did not meet the diabetes criteria. To allow for this three-year screening period, screening-based incidence rates were calculated annually for 2006–2012 because laboratory data on serum glucose and HbA1c tests were only reliably available from 2003 onwards in the Clalit database. *“Previously-unscreened incidence”* were diabetes cases identified among only the members who were not screened in the previous three years and who had no indication of diabetes at the beginning of the year from 2006–2012. All rates of change were evaluated from 2006–2012 to allow for comparisons between parameters, and statistical significance was tested using linear regression.

## Results

The Clalit population aged 26 years and older in 2004 consisted of 2,110,824 members, and in 2012 of 2,379,712 members (a 12.7% increase over the entire study period and a 9.7% increase from 2006 to 2012) (Table 1). There were 226,855 diabetes cases in 2004 (10.7% of the Clalit population age 26 and older) compared to 343,554 cases in 2012 (14.4% of the Clalit population), with a cumulative 34.3% increase in the crude prevalence rate over nine years and a 19.1% increase during the period from 2006 through 2012. In 2012, there were 52.2% women in the Clalit population and the mean age was 50.3 ± 17.3. In comparison, the subpopulation with diabetes in the same year comprised 50.8% women and the mean age of the population with diabetes was 65.5 ± 13.1.

**Table 1:** **Diabetes prevalence and mortality from 2004–2012**

	**2004**	**2005**	**2006**	**2007**	**2008**	**2009**	**2010**	**2011**	**2012**	**Change 2006-2012** ^**b**^
Total adult population^a^	2110824	2137856	2170220	2199626	2229850	2263441	2296171	2338708	2379712	9.7%
Prevalent cases	226855	246434	263135	279240	294117	308042	321091	335232	343554	30.6%
Crude prevalence (×1,000)	107.5	115.3	121.2	126.9	131.9	136.1	139.8	143.3	144.4	19.1%
Age-standardized prevalence (×1,000)	93.5	100.1	105.2	109.9	114.1	117.6	120.5	123.4	124.1	18.0%
Prevalence by age group (×1,000)										
26-34y	7.3	7.9	8.4	8.8	9.1	9.3	9.3	9.3	9.4	11.9%
35-44y	31.4	33.9	35.4	36.4	37.0	37.1	36.6	35.8	34.5	-2.5%
45-54y	89.5	95.3	100.0	103.9	107.0	109.9	112.2	114.6	114.4	14.4%
55-64y	176.5	186.7	194.8	202.5	210.1	217.0	223.5	229.6	231.0	18.6%
65-74y	251.2	267.8	281.8	295.9	308.2	317.1	324.9	332.1	333.9	18.5%
75-84y	235.3	259.2	278.6	298.5	317.0	333.9	348.5	363.1	372.2	33.6%
85y+	152.8	175.1	195.0	215.0	231.9	251.0	266.6	282.0	294.7	51.1%
Age-standardized all-cause mortality of members with diabetes (×1,000)	13.8	12.4	12.2	12.1	11.6	10.9	10.7	10.5	10.7	-12.3%
Mortality by age group (×1,000)										
26-34y	3.4	1.8	1.6	2.8	2.8	1.4	2.4	2.1	1.7	6.3%
35-44y	3.6	2.3	3.5	3.2	2.5	3.1	3.6	2.7	3.0	-14.3%
45-54y	7.3	7.0	6.5	6.8	6.5	6.1	5.7	6.0	6.4	-1.5%
55-64y	14.9	13.6	13.4	13.3	13.3	12.2	11.4	11.2	12.0	-10.4%
65-74y	34.0	31.1	30.8	28.7	28.0	26.9	25.9	25.7	26.0	-15.6%
75-84y	81.0	79.0	74.0	73.0	69.8	66.5	65.2	64.3	64.8	-12.4%
85y+	222.5	217.7	210.9	211.6	195.8	186.1	179.4	185.5	185.2	-12.2%

^a^For Clalit members aged 26 and older ^b^Percent change for years 2006 and 2012 was calculated in order to compare with screening-based incidence data starting in 2006.

**Figure 1:**
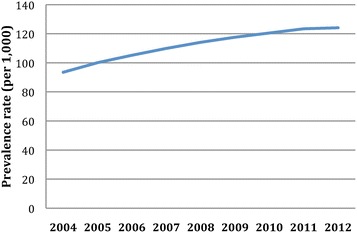
**Trends in age-standardized prevalence of diabetes from 2004 to 2012.**

**Figure 2:**
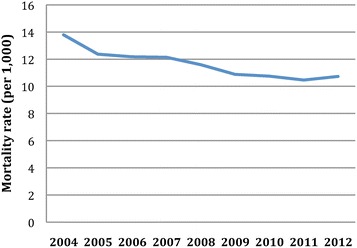
**Trends in age-standardized all-cause mortality among the diabetes population from 2004 to 2012.**

Trends in diabetes prevalence and mortality of the diabetes population are presented in Table 1 and Figures 1 and 2. The age-standardized prevalence of diabetes in the study population increased 18.0% between 2006 and 2012 from 105.2/1000 to 124.1/1000. This trend was statistically significant with p < 0.0001. The increase in prevalence was more marked in the older age groups, with the greatest rise in prevalence in those aged 85 and older, nearly doubling over the study period. While prevalence rose overall, the annual rate of increase continuously slowed over the study period (Figure 1). This leveling in prevalence rates is seen among all age groups, particularly in those under age 65 for whom a plateauing rate is observed from 2009 to 2012 (Table 1). Simultaneously, during the seven-year period from 2006–2012, the age-adjusted mortality rates among the diabetes population fell from 12.2/1000 to 10.7/1000, a relative decline of 12.3% (p = 0.0002). The age-stratified trends reveal that there was a gradual and consistent decline in mortality among adults aged 45 and older. The decline in mortality in diabetics is highly correlated to the mortality in the general Clalit population (−11.8% over the same period, p <0.001) (results not shown).

**Figure 3:**
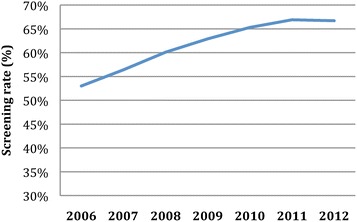
**Trends in age-standardized annual screening rate among the at-risk population from 2006 to 2012.**

Annual screening rates and incidence trends from 2006 to 2012 are presented in Table 2, examining overall incidence and incidence in subpopulations defined by recent screening. While the annual proportion of screened individuals, out of previously-unscreened non-diabetics, increased from 53.0% in 2006 to 66.7% in 2012 (p = 0.0004) (Table 2 and Figure 3), the absolute number of previously-unscreened at-risk individuals declined from 576,895 in 2006 to 411,858 in 2012 (−28.6%). A decline in the age-adjusted incidence rate was observed from 13.3/1000 in 2006 to 10.8/1000 in 2012 (p = 0.0002), with the total at-risk population growing marginally (5.9%) and the annual number of incident cases shrinking over the study period.

**Table 2:** **Diabetes incidence and screening from 2004–2012 for total population parameters and from 2006–2012 for screening-based parameters**

	**2004**	**2005**	**2006**	**2007**	**2008**	**2009**	**2010**	**2011**	**2012**	**Change 2006-2012**
**Total population** ^**a**^ **parameters**										
Total at-risk population^a^ at beginning of year	1891846	1883940	1891392	1907065	1920369	1935712	1955387	1975066	2003464	5.9%
Incident cases	30392	28648	26680	26405	25942	25191	24703	26110	21776	-18.4%
Crude incidence ( 1,000)	16.1	15.2	14.1	13.8	13.5	13	12.6	13.2	10.9	-22.7%
Age-standardized incidence (×1,000)	14.8	14.2	13.3	13.1	12.9	12.6	12.3	13	10.8	-18.8%
Incidence by age group (×1,000)										
26-34y	2.2	2	1.9	1.9	1.7	1.7	1.6	1.6	1.6	-15.8%
35-44y	7	6.7	6.2	6	5.6	5.6	5.1	5.2	4.6	-25.8%
45-54y	16.2	15.7	15.2	14.3	14.6	14.3	14.5	15.1	13	-14.5%
55-64y	27.1	25.7	24.2	24	23.7	23.3	23.1	24.1	20	-17.4%
65-74y	34.1	32.6	29.7	30.4	30.1	28.5	27.7	29.9	23	-22.6%
75-84y	30.4	29	26.6	27.4	26.4	25.6	24.3	27	21.1	-20.7%
85y+	21.7	19.6	18.7	19.3	19.1	18.1	17.1	18.1	16	-14.4%
**Screening-based parameters**										
Annual screening rate^b^			53.0%	56.4%	60.1%	62.9%	65.3%	66.9%	66.7%	25.8%
Previously-screened (prior 3 years) at-risk population^a^			1314497	1362188	1411353	1466190	1520518	1562205	1591606	21.1%
Previously-screened incident cases (only those with previous negative screening)			20643	20794	21105	20797	20799	22143	17934	-13.1%
Crude previously-screened incidence (×1,000)			15.7	15.3	15.0	14.2	13.7	14.2	11.3	-28.2%
Age-standardized previously-screened incidence (×1,000)			13.2	12.9	12.8	12.3	12.1	12.6	10.2	-23.0%
Previously-unscreened (prior 3 years) at-risk population^a^			576895	544877	509016	469522	434869	412861	411858	-28.6%
Previously-unscreened incident cases			6037	5611	4837	4394	3904	3967	3842	-36.4%
Crude – previously-unscreened incidence (×1,000)			10.5	10.3	9.5	9.4	9.0	9.6	9.3	-10.9%
Age-standardized previously-unscreened incidence (×1,000)			14.5	14.9	14.2	14.6	14.2	15.7	15.1	4.0%

^a^For Clalit members aged 26 and older.

^b^Patients, age 26 and older, that received an HbA1c or glucose test in a given year, out of the previously-unscreened (prior 3 years) at-risk population.

**Figure 4:**
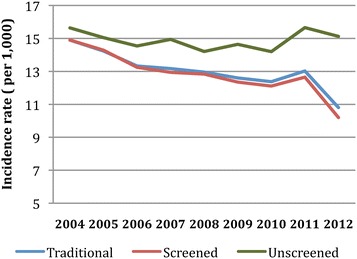
**Trends in age-standardized incidence of diabetes from 2004 to 2012.**

This downward trend in the incidence rate was interrupted by a relatively sharp increase in 2011 followed by a sharp decrease in 2012 (Figure 4). The age-stratified incidence rates showed similar trends of a steady and slow decline across the age groups, with the exception of a slight increase in new cases in 2011, particularly in the 65–84 age groups. Among the previously-screened at-risk population, there was a decline in incidence from 2006 to 2012, with rates of 13.2/1000 and 10.2/1000, respectively (p = 0.0007) (Table 2). The same increase and decrease over 2011–2012 was observed in the previously-screened incidence rate (Figure 4). The previously-unscreened incidence rates remained relatively constant over the 2006–2012 period (p = 0.267) (Figure 4).

## Discussion

In the present study we retrospectively apply a composite diabetes case-finding algorithm in an EMR database of a large diverse population and examine several population-based parameters that inform the trends of diabetes prevalence. During the study period from 2004 to 2012, there was a consistent but gradually diminishing upward trend in diabetes prevalence, with a crude cumulative increase of 34.3% over nine years. Our findings demonstrate that the trends in prevalence are driven by a relative balance between detection, the emergence of new cases, and increased survival among diabetes patients. Contrary to the view that there is an ensuing local diabetes “epidemic”—parallel to the global “pandemic”—we observe that drivers in diabetes trends are moving toward equilibrium, with a decline in the number of “new incident” cases (among previously-screened individuals), a slowing in the number of “newly-detected” cases (previously-unscreened), and declining mortality rates among diabetes patients. The most striking of these trends is that the recorded incidence of new diabetes cases has decreased by nearly 23% over the nine-year period, with an even sharper decline among the at-risk subpopulation that has been previously screened and did not have diabetes when screened in the past. Whether these trends are sustained will dictate if the decade-long upward trend in diabetes continues to slow or plateau.

The overall increase in prevalence of diabetes observed in the study population is consistent with prevalence reports from other countries; however, the rate of increase is slowing in our population compared to the escalating trends reported in previous research [2,4-7,14]. A majority of these studies have concluded that increasing prevalence is attributable to declining mortality and rising annual incidence [5-8]. Similar to these studies, mortality rates among diabetes patients in our study population are gradually declining, but unlike their findings, overall incidence rates are also declining. Much of the increase in prevalence in our population over the period of this study is occasioned by intensified screening and case finding, as well as the decline in mortality. Among the previously-unscreened population at risk, incidence rates remain fairly constant, but the overall number of these “newly-detected” cases is declining as the pool of unscreened patients is gradually depleted. The decline in these newly-detected cases is occurring despite an increase in the rates of annual screening among those at-risk and previously-unscreened, which partly explains past upward trends and current leveling of the prevalence.

The relationship between screening and its impact on prevalence is further confirmed when looking at the incidence trends in 2011 to 2012 (Figure 4). In 2011, an organization-wide intensive case-finding program for diabetes and pre-diabetes was launched in Clalit. This likely explains the sudden rise and resultant drop of incident cases observed from 2011–2012. Further analysis revealed that this jump was predominantly a consequence of the number of HbA1c tests conducted among the at-risk population, substantiating the direct positive association between screening intensity and incidence rates. In an organization like Clalit that is a payer and provider with long-term—or even lifetime—membership, there are interventions implemented on various levels that potentially prevent the onset of diabetes (thereby, lowering incidence) and possibly extend the lifespan of patients with diabetes (thereby, lowering mortality rates). These interventions can also be contributing factors to the observed trends.

Several limitations are to be considered in our study. We were not able to quantify all key factors contributing to the trends in incidence, mortality, and screening rates due to missing or incomplete data on body mass index, physical activity, and diet in our dataset. Consequently, while we cannot describe potential changes in behavioral patterns, it is not expected that the risk factors or lifestyle variables would have a direct impact on prevalence beyond their impact on the three key components examined in this study. Additionally, this study is intended to focus on type 2 diabetes mellitus; however, due to data limitations, we could not distinguish between type 1 and type 2 diabetes in our dataset. In order to minimize the impact of this limitation in the population we defined, we have restricted the study sample to include only adults over the age of 25, thus eliminating the majority of members with type 1 diabetes from our sample. Restricting the sample in this way would exclude most individuals with type 1 diabetes from calculations of incidence, since type 1 is usually diagnosed at younger ages. Furthermore, because there is no indication in the Clalit laboratory data whether a given glucose test was done in a fasting state, some of our criteria used two parameters to augment the diagnostic criteria possibilities. Using two parameters resulted in an algorithm with relatively higher specificity than sensitivity, which may have underestimated the absolute incidence and prevalence rates; however, this should not have had a significant effect on the trends over time. Finally, the “previously-unscreened” incidence fails to identify patients who were not screened but did develop new onset diabetes in the year being studied.

The findings in our study may be uniquely identified in a closed provider-payer system with an unusually stable and universally-insured population, but nevertheless reveals that much of the increase in prevalence results from improved case finding and declines in mortality, rather than from persistent and escalating increases in the onset of disease.

## Conclusions

In conclusion, we observe that the rise in diabetes prevalence is decelerating and incidence is declining—most evident among the “previously-screened” subgroup—despite aging of the population and an expansion of testing among at-risk members. The stability of Clalit’s patient population, with low membership attrition from year to year, and the standardization of data reporting in EMRs used across all care settings within the organization enables consistency and completeness of the data, as well as providing the ability to track the impact of policy changes on case finding. Although there is currently a lack of uniformity in how measures of prevalence and incidence are constructed across studies due to variations in data availability, we maintain that with the increasing uptake of integrated EMRs in the United States and Europe, there will be a shift toward more standardized and uniform measures of disease burden. While others have shown diabetes prevalence to be continuously rising, attributable to both declines in mortality and increases in annual incident cases, our results suggest that increasing diabetes screening rates (with attenuating impact due to the diminishing pool of “previously-unscreened” individuals) coupled with declines in mortality are driving the increase in prevalence, but an expansion in incident cases is not observed. With incidence rates greatly influenced by organizational policies for screening, there is a need to consider organizational screening guidelines when comparing prevalence and incidence trends across settings and contexts.

## Additional files

Additional file 1: Figure S1. Algorithm for Diabetes Registry. The eight identified criteria were consolidated by lumping lab tests together to form six criteria representing the most robust criteria for identifying diabetics in the Clalit electronic database. Additional file 1: Figure S1 illustrates the process and order in which each of the six criteria was applied to the electronic database to establish the composite definition of diabetes. We applied the most specific criteria first to create the hierarchy with a total of 480,295 patients identified and included in the diabetes registry. Additional file 2: Table S1. Diabetes Prevalence and Incidence Final PHM July 27 2014. Internal validation of diabetic entry criteria with subsequent confirmation. In order to determine which diagnostic criteria we would use to identify members with diabetes, we first applied a modified version of the current diagnostic criteria as defined by the American Diabetes Association (ADA) to the Clalit database. This includes abnormal glucose tests (a. glucose ≥126 mg/dl (7 mmol/l) in a fasting patient; b. glucose ≥200 mg/dl (11.1 mmol/l) on a casual blood test; c. HbA1c ≥6.5 (7.8 mmol/l)) confirmed by repeated test within 3–6 months. Because there is no indication in the Clalit laboratory data whether a given glucose test was done in a fasting state, we adapted from the ADA the parameters of glucose >200 mg/dl and HbA1c ≥6.5. We then added to these two lab parameters both diabetes diagnoses and medications to augment the diagnostic criteria possibilities. Using these four parameters we generated 10 criteria, as shown in this table, as case definitions of diabetes to identify from the Clalit electronic database potential diabetics between 2004–2012. We then cross-tabulated the entry criteria of each potential diabetic to determine whether they had any of the 10 subsequent confirmation criteria in the following three years to conduct an internal validation. We singled out those entry criteria where members were more than 80% likely on average (mean%) to have all nine confirmation criteria in the subsequent three years. As indicated in the table (rows 3 and 4) HgbA1c > = 6.5 (×2) and Glucose > = 126 (×2) had relatively low levels of subsequent corroboration over time (internal validation) and were therefore not included in the final algorithm. Additional file 3: Table S2. Progressive aggregation of cases based on the hierarchy of criteria used to identify diabetics. The table shows the total number of cases identified by each criterion displayed in Additional file 1: Figure S1, as well as the number of new cases that are added by each.

## Abbreviations

CDC: Centers for Disease Control and Prevention
Clalit: Clalit Health Services
NHANES: National Health and Nutrition Examination Survey
EMR: Electronic medical record
