# Supplementary material for: Towards a subsiding diabetes epidemic: trends from a large population-based study in Israel
Source: Popul Health Metr. 2014 Oct 30;12:32. doi: 10.1186/s12963-014-0032-y (PMC4233034; doi:10.1186/s12963-014-0032-y)
Supplement: Additional file 1: Figure S1. — Algorithm for Diabetes Registry. The eight identified criteria were consolidated by lumping lab tests together to form six criteria representing the most robust criteria for identifying diabetics in the Clalit electronic database. Additional file 1: Figure S1 illustrates the process and order in which each of the six criteria was applied to the electronic database to establish the composite definition of diabetes. We applied the most specific criteria first to create the hierarchy with a total of 480,295 patients identified and included in the diabetes registry. [file 12963_2014_32_MOESM1_ESM.pdf]

Figure 1s. Algorithm for Diabetes Registry

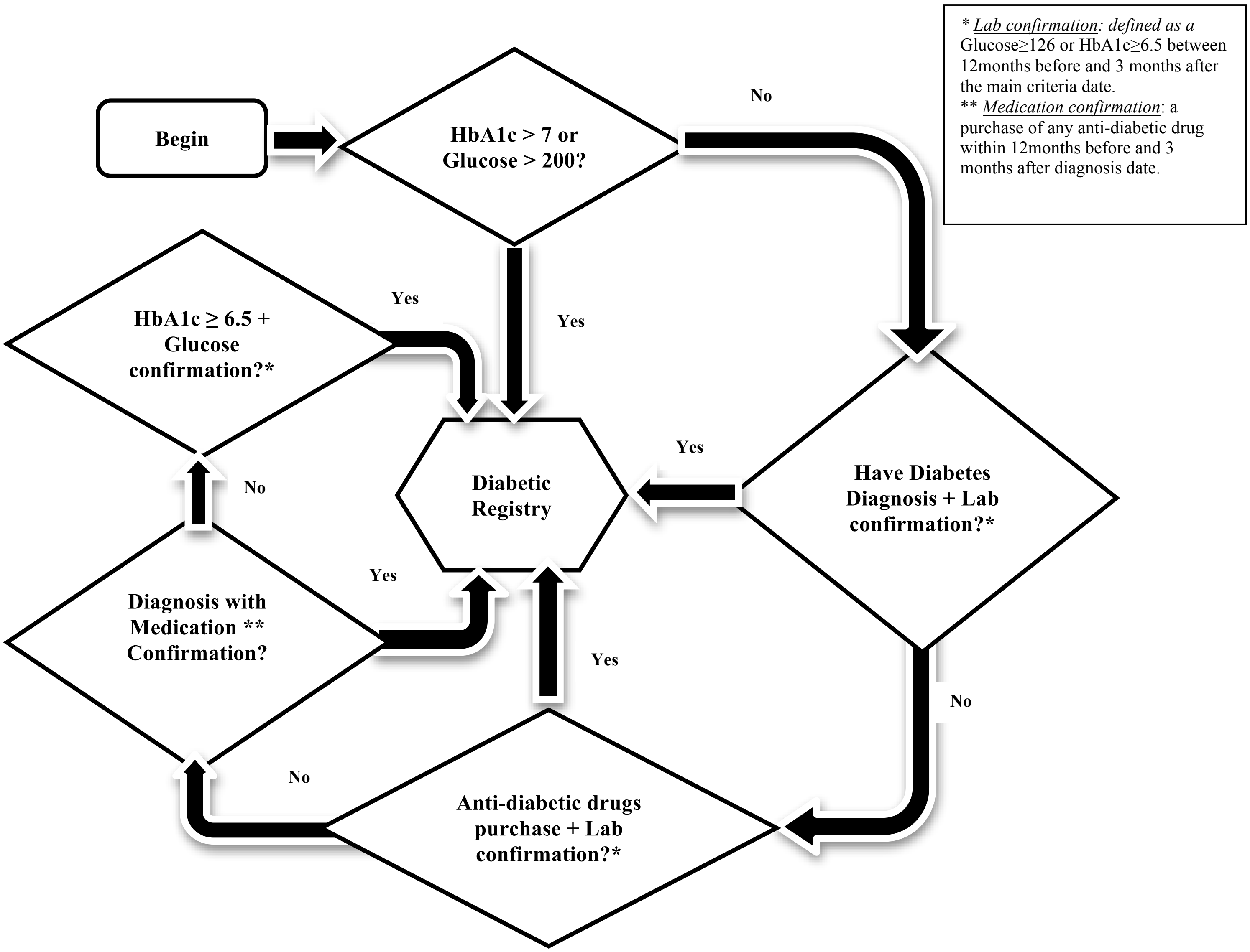

The eight identified criteria were consolidated by lumping lab tests together to form six criteria representing the most robust criteria for identifying diabetics in the Clalit electronic database. Figure 1s illustrates the process and order in which each of the six criteria was applied to the electronic database to establish the composite definition of diabetes. We first applied the most specific criteria first to create the hierarchy with a total of 480,295 patients identified and included in the diabetes registry.
