# Supplementary material for: Towards a subsiding diabetes epidemic: trends from a large population-based study in Israel
Source: Popul Health Metr. 2014 Oct 30;12:32. doi: 10.1186/s12963-014-0032-y (PMC4233034; doi:10.1186/s12963-014-0032-y)
Supplement: Additional file 2: Table S1. — Diabetes Prevalence and Incidence Final PHM July 27 2014. Internal validation of diabetic entry criteria with subsequent confirmation. In order to determine which diagnostic criteria we would use to identify members with diabetes, we first applied a modified version of the current diagnostic criteria as defined by the American Diabetes Association (ADA) to the Clalit database. This includes abnormal glucose tests (a. glucose ≥126 mg/dl (7 mmol/l) in a fasting patient; b. glucose ≥200 mg/dl (11.1 mmol/l) on a casual blood test; c. HbA1c ≥6.5 (7.8 mmol/l)) confirmed by repeated test within 3–6 months. Because there is no indication in the Clalit laboratory data whether a given glucose test was done in a fasting state, we adapted from the ADA the parameters of glucose >200 mg/dl and HbA1c ≥6.5. We then added to these two lab parameters both diabetes diagnoses and medications to augment the diagnostic criteria possibilities. Using these four parameters we generated 10 criteria, as shown in this table, as case definitions of diabetes to identify from the Clalit electronic database potential diabetics between 2004–2012. We then cross-tabulated the entry criteria of each potential diabetic to determine whether they had any of the 10 subsequent confirmation criteria in the following three years to conduct an internal validation. We singled out those entry criteria where members were more than 80% likely on average (mean%) to have all nine confirmation criteria in the subsequent three years. As indicated in the table (rows 3 and 4) HgbA1c > = 6.5 (×2) and Glucose > = 126 (×2) had relatively low levels of subsequent corroboration over time (internal validation) and were therefore not included in the final algorithm. [file 12963_2014_32_MOESM2_ESM.pdf]

Table S1. Internal validation of diabetic entry criteria with subsequent confirmation

**Criteria***	# of Patients	HbA1c > 7.0	Glucose > 200	HbA1c >= 6.5 (x2)	Glucose >= 126 (x2)	HbA1c >=6.5 + Glucose >=136	Dx + Glucose >= 126	Dx + HbA1c >= 6.5	Med + Glucose >= 126	Med + HbA1c >= 6.5	Dx + Med	Mean %
HbA1c > 7.0	334,233	---	58.5%	100.0%	96.7%	94.7%	91.9%	89.7%	93.9%	90.9%	89.9%	**89.6%**
Glucose > 200	219,919	88.9%	---	92.1%	100.0%	87.7%	97.2%	91.3%	89.6%	93.1%	89.9%	**92.2%**
HbA1c >= 6.5 (x2)	453,090	73.8%	44.7%	---	87.1%	76.5%	80.7%	76.2%	80.5%	75.1%	76.5%	74.6%
Glucose >= 126 (x2)	621,225	52.0%	20.9%	63.6%	---	55.8%	55.4%	61.3%	55.7%	59.2%	56.9%	53.4%
HbA1c >=6.5 + Glucose >=136	346,485	87.3%	55.7%	100.0%	100.0%	---	91.3%	92.0%	91.9%	91.0%	88.3%	**88.6%**
Dx + HbA1c >= 6.5	365,766	78.8%	52.8%	100.0%	94.1%	86.5%	---	91.1%	92.2%	86.7%	91.6%	**86.0%**
Dx + Glucose >= 126	380,519	78.8%	52.8%	90.7%	100.0%	83.7%	87.6%	---	84.7%	88.8%	89.2%	**84.0%**
Med + HbA1c >= 6.5	364,528	86.1%	54.1%	100.0%	95.0%	87.4%	92.6%	88.4%	---	92.4%	93.9%	**87.8%**
Med + Glucose >= 126	367,594	82.7%	55.7%	92.6%	100.0%	85.8%	86.3%	92.0%	91.7%	---	93.0%	**86.6%**
Dx + Med	379,490	79.1%	52.0%	91.3%	93.1%	80.6%	88.2%	89.4%	90.2%	90.1%	---	**83.8%**

*In criteria with two parameters, the second parameter had to be within 12 months before or 3 months after the first parameter.

In order to determine which diagnostic criteria we would use to identify members with diabetes, we first applied a modified version of the current diagnostic criteria as defined by the American Diabetes Association (ADA) to the Clalit database. This includes abnormal glucose tests (a. glucose ≥ 126 mg/dl (7mmol/l) in a fasting patient; b. glucose ≥ 200 mg/dl (11.1mmol/l) on a casual blood test; c. Glucose >= 126 X2, d. HbA1c ≥ 6.5 (7.8mmol/l)) confirmed by a repeated test within 3-6 months) [1]. Because there is no indication in the Clalit laboratory data whether a given glucose test was done in a fasting state, we adapted from the ADA the parameters of glucose >200 mg/dl and HbA1c ≥ 6.5. We then added to these two lab parameters both diabetes diagnoses and medications to augment the diagnostic criteria possibilities. Using these four parameters we generated ten criteria (Table 1s) as case definitions of diabetes to identify from the Clalit electronic database potential diabetics between 2004 -2012. We then cross-tabulated the entry criteria of each potential diabetic to determine whether they had any of the other nine subsequent confirmation criteria in the following three years, to conduct an internal validation. We singled out those entry criteria where members were more than 80% likely on average (mean %) to have all nine confirmation criteria (See Table 1s) in the subsequent three years. As indicated in the above table (rows 3 and 4) HgbA1c >= 6.5 (x2) and Glucose >= 126 (x2) had relatively low levels of subsequent corroboration over time (internal validation) and were therefore not included in the final algorithm.
