# Supplementary material for: Towards a subsiding diabetes epidemic: trends from a large population-based study in Israel
Source: Popul Health Metr. 2014 Oct 30;12:32. doi: 10.1186/s12963-014-0032-y (PMC4233034; doi:10.1186/s12963-014-0032-y)
Supplement: Additional file 3: Table S2. — Progressive aggregation of cases based on the hierarchy of criteria used to identify diabetics. The table shows the total number of cases identified by each criterion displayed in Additional file 1: Figure S1, as well as the number of new cases that are added by each. [file 12963_2014_32_MOESM3_ESM.pdf]

Table S2. Progressive aggregation of cases based on the hierarchy of criteria used to identify diabetics

**Case Finding Criteria**	# of cases	Newly added cases
Very High Lab test	358,524	358,524
Dx + Lab test	412,961	88,121
Med + Lab test	395,200	13,887
Dx + Med	379,490	13,065
HbA1c + Glucose	346,485	6,698
**Total in Registry**		**480,295**

The table above shows the total number of cases identified by each criterion displayed in the figure above as well as the number of new cases that are added by each.
